# A New Golden Age? Proposal for an Innovative Global Health Funding Mechanism for Middle‐Income Countries

**DOI:** 10.1002/gch2.201700015

**Published:** 2017-09-21

**Authors:** Claire Chaumont, Jenny Hsi, Christine Bohne, Sana Mostaghim, Suerie Moon

**Affiliations:** ^1^ Harvard T.H. Chan School of Public Health Harvard University 677 Huntington Ave Boston MA 02115 USA

## Abstract

Despite economic growth and increased global commitment to health financing in the past decades, the health needs of some of the world's most vulnerable people remain overlooked. In particular, middle‐income countries (MICs) often face the conundrum of receiving reduced development assistance for health (DAH) while still being home to most of the world's poor and the majority of the global burden. We believe that this reflects shortcomings in the global DAH system's architecture, which operate on principles that do not respond well to current realities. Hence, we propose a novel mechanism for international health financing and action that specifically addresses the newly emerged strengths and needs of MICs. The Incentives for Health (I4H) Alliance will offer MICs flexible incentives in exchange for their making and meeting health‐related commitments in their countries. Countries can set their own health targets, in alignment with the existing Sustainable Development Goals' framework, and those that achieve them will be subsequently rewarded with financial or other incentives, which are not restricted to the health sector. We believe that the I4H Alliance will promote greater MIC involvement towards global health financing both as incentive providers and recipients; encourage collaboration between Ministries of Health and Finance; and provide a needed complement to traditional DAH mechanisms. We advocate for the creation of I4H at a MICs‐oriented financing institution such as the New Development Bank. We intend I4H to spark new thinking around innovative health financing approaches to ensure that the “golden age” of global health remains ahead.

## 1 Introduction

**Figure 1:**
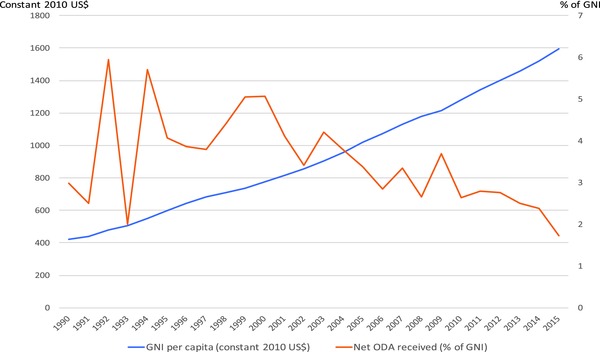
Trends in GNI per capita and Net ODA as a percentage of GNI received by Vietnam between 1990 and 2015.^1^

Between 1990 and 2015, Vietnam, a middle‐income country (MIC) by the World Bank classification, experienced a steady increase in its gross national income (GNI) per capita (in constant 2010 US$) by more than three‐fold. At the same time, the net Overseas Development Assistance (ODA) it received as a percentage of its GNI was slowly decreasing. Yet from year to year, this latter figure varied dramatically, with a temporary dip of more than 4 percentage points in 1993, and a decrease from 5.7% in 1994 to 3.8% only three years later (**Figure** **1**).

What might explain these important variations? The allocation of foreign aid often results from a wide variety of reasons, including the needs of the recipient country, but also historical relations with donor countries, peer effects, the country's external debts, and its political behavior in international forums.^2^, ^3^, ^4^

That all these factors contribute to differential treatment between countries is commonly accepted as a global political reality. However, the complexity of this resource allocation process undoubtedly raises issues of fairness and efficiency. Indeed, many have criticized the lack of transparency and effectiveness of the complex decision calculus negotiated between donor countries, recipient countries, and myriad global institutions for aid.^5^, ^6^

Development assistance for health (DAH) is a good example of the shortcomings of the current system. In this sector, one topic in particular has received much attention: the relationship between the recipient country's economic status and its health needs. While poverty and health are intrinsically connected, national income is often a poor proxy for disease burden, in part because of health and income inequalities within countries. Since 2000, the number of low‐income countries worldwide has fallen from 70 to just 31.^7^, ^8^ As a result, a new geography of poverty has emerged: today, over 3/4's of the world's poorest people resides in MICs.^9^ Yet despite accounting for over 80% of the global disease burden, MICs only receive 59% of global DAH.^10^ The distribution of this “new bottom billion” of poor individuals in increasingly richer MICs creates new challenges for DAH.^11^

Meanwhile, an assumption remains in the global health and aid system that rising MICs will become more able to take care of their populations' needs through increased domestic health spending. This is not baseless or unreasonable. For example, between 2000 and 2013, countries in the Southeast Asia, East Asia, and Oceania region leveraged their economic growth from previous decades to increase government health expenditures by 13.4% annually.^12^ But a blanket statement linking economic and human development can also result in unstable funding for health and a deepening of subnational inequalities. This can be seen in the recent decisions of several multilateral donors to start exiting from countries with rising national income deemed no longer in need of external funding. For a country such as Vietnam, however, Salvado et al. have estimated that it stands to lose 36.5% of current Official Development Assistance (ODA) between 2015 and 2030, due to its combined “graduation” from the World Bank's International Development Association, GAVI, DfiD, and the Asian Development Fund.^7^ These shocks to a country's funding streams can have substantial impact on health services delivery and thus health outcomes. If not considered properly, the “middle‐income conundrum” threatens to trap some of the world's “new bottom billion” in unmet need and poverty.

While a number of initiatives have burgeoned to address the shortcomings of current global health financing mechanisms, we believe stronger action is needed to challenge the status quo. Hence, this paper seeks to spark new thinking around how to best fund health in MICs. We propose a novel mechanism for international health financing and action that specifically addresses the newly emerged strengths and needs of MICs, in context of the changing global landscape for aid financing and governance. We term this mechanism the Incentives for Health (I4H) Alliance, which offers MICs flexible incentives in exchange for their making and meeting health‐related commitments in their countries. Countries can set their own health targets under the I4H Alliance's broad, SDG‐based framework, and those that achieve their targets will be rewarded with financial or other incentives which are not restricted to the health sector. We believe that the I4H Alliance will promote greater MIC involvement towards global health financing, both as incentive providers and as recipients, as well as provide a needed complement to traditional DAH mechanisms.

## 2 Health and Aid in Middle‐Income Countries: What has Changed?

Since 1990, global DAH disbursement has more than tripled, plateauing in the last several years at approximately $36 billion USD annually.^12^ DAH now constitutes over 25% of all official development assistance (ODA). Despite these tremendous investments, unmet health needs worldwide – and especially in MICs – remain enormous. This mismatch can be attributed to a design challenge: that the existing global DAH system operates on principles that do not respond well to current realities.

In the last 30 years, significant worldwide growth in national populations and economic capacities have challenged the traditional orientation of foreign aid from higher‐income to lower‐income countries.^13^, ^14^, ^15^, ^16^, ^17^ These shifts bring to light the accountability and governance challenges already at play in the current system.^5^ It also deepens the mismatch between resources needed and allocated for addressing health in MICs. Countries' eligibility for foreign aid often remain linked to their GNI per capita capita (GNIpc) or their World Bank lending group designation. According to Ottersen et al., while 13 out of 14 multilateral and bilateral donors currently use GNIpc as an explicit criterion to allocate resources, none incorporates a criterion based on sub‐level economic or health inequalities, such as the Gini coefficient, inequality in health expectancy (LEi), or the Inequality‐adjusted Human Development Index (IHDI).^6^, ^7^, ^8^, ^9^, ^10^, ^11^, ^12^, ^13^, ^14^, ^15^, ^16^, ^17^, ^18^ Other multilateral donors have similarly overlooked the role of in‐country inequalities to determine DAH “graduation” criteria.^7^, ^19^, ^20^ (GFTAM, 2016)^21^ For example, China and Indonesia – home to more than 20% of the world's poor – are currently no longer eligible for IDA loans, the World Bank's most favorable concessionary lending for poorer countries.^11^, ^22^

## 3 Existing Efforts at Addressing DAH Challenges for Middle‐Income Countries

A number of policy analyses and initiatives have recently sought to significantly reform the frameworks for global cooperation and governance around DAH, to better address the above challenges.

First, efforts have been made to establish new global norms around who should contribute or receive how much and for what ends. One such example is the 2014 Chatham House report, which delineates the responsibilities of countries towards domestic, external and joint international financing and attempts to establish what amount of international financing should be dedicated to health. (Rottingen et al.)^22^ Experts have also long called for the creation or strengthening of global health governance structures, using a normative approach. Recently, the Framework Convention on Global Health and the collective rights framework have attracted attention for their efforts to propose structures to more effectively regulate and enforce DAH activities.^24^, ^25^, ^26^

Besides, there have been several attempts at refining the eligibility criteria linked to overseas development assistance. The United Nations' Least Developed Countries categorization, the Human Development Index or the Social Progress Index all attempt to incorporate human and social measurements alongside traditional economic considerations for priority‐setting and resources allocation.^27^, ^28^, ^29^ More recently, the Global Fund led a multi‐partner initiative, the Equitable Access Initiative, tasked to redefine country eligibility criteria, to ultimately better guide DAH investments.^21^ The initiative ultimately recommended the adoption of a multi‐criteria framework, accounting not only for resources, but also local health needs relative to income, government's investment priorities, and equity metrics for resource allocation (EAI, 2016).^21^

The breadth of current discussions around DAH reveals a clear acknowledgment of the acute need of reform of the current system. Initiatives such as the EAI can also help develop policy tools to practically reform the way DAH is allocated. However, despite their immediate applicability, these initiatives taken as a whole insufficiently address the middle‐income conundrum described above. In particular, few proposals explicitly consider; a) the tension faced by many MICs between investing in health vs. other sectors more directly relevant to their short‐term economic growth; b) the integration of financial flows for health with other health‐related transfers (intellectual property, technical expertise), which are sometimes more directly needed by MICs; and c) the need to explicitly combine better DAH accountability with MIC's rising calls for more autonomy and influence on the global stage.

## 4 I4H Alliance: A New Mechanism to Stimulate Health Investments in Middle‐Income Countries

**Figure 2:**
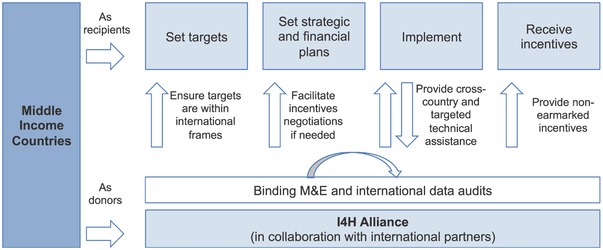
Operational schematic for the Incentives for Health (I4H) Alliance.

This analysis leads us to propose the creation of a novel mechanism, the Incentives for Health (I4H) Alliance, to increase international and domestic health financing in complement to existing DAH flows (**Figure** **2**). In contrast to traditional financing vehicles such as grants and loans, the I4H model offers countries – particularly MICs – flexible incentives in exchange for their making and meeting domestic health‐related commitments. We envision I4H to operate through the following principles:

1. Participant countries each voluntarily design their own health targets in alignment with the Sustainable Development Goals (SDGs). The definition of health targets will follow these principles:

- A country's targets must be as ambitious or more ambitious than the agreed‐upon SDGs. This helps maintain coherence with previously existing agreements.
- Recognizing that not all countries can achieve the SDGs at the same speed, targets may be developed only for sub‐groups of the population – as long as targeting these sub‐groups can improve health equity. For example, if one country decides to develop a target based on maternal mortality, it can decide to target the poorest segment of the population only. Assessing how targets can improve health equity can be done using recommendations developed by the EAI.
- The time frame of the target is adjusted based on the country's current health situation, to ensure targets are achievable within the time frame proposed.
- Targets – and the incentives linked to them – are assessed based on two complementary measures: the number of DALYs averted through achieving the target, and its impact on health equity.
- Targets must be linked to a detailed implementation plan and corresponding resource mobilization plans. Countries may finance the project through any means they wish, although domestic sources are strongly encouraged, in order to promote country ownership and sustainability.

2. Reporting on progress is compulsory. Indicators and reporting mechanisms are standardized based on the current SDGs' M&E framework. Participant countries must also agree to periodically receive international teams to audit their data. Specific grants can be made available for countries to improve information systems. Apart from the binding reporting obligation, a participant country is free to implement the health improvement projects however it deems fit.

3. Finally, once a participant country achieves its health targets, it gains access to specific, pre‐negotiated incentives. two scenarios are possible:

- In a more advanced model, we propose to widen the scope of incentives proposed as part of the I4H Alliance, to develop customized packages of incentives. In a simplified model, incentives take the form of non‐earmarked preferential loans from a development financing institution. These loans are designed to have interest rates below what the country can expect to access in commercial markets or existing aid mechanisms, and come with only a loose set of social and environmental conditionalities. In addition, loans are made commensurate to the size of the domestic investment needed to achieve the goal, in such a way that the total value of the incentive is higher than the costs linked to setting up and operating the changes needed to achieve the target. The size of the loan is also related to the health impact attributed to achieving the target, considering the number of DALYs adverted and its impact on health equity. An important characteristic of the I4H design is that the loan is conceived as a reward for achieving health goals, and does *not* itself need to be used towards health – it can be flexibly deployed for whatever ends the country deems as a priority (e.g. public works, education, economic development projects, etc.).
- In an advanced model, customized incentive packages may be developed for each country. MICs are a diverse group with different needs and interests. For example, upper‐middle‐income countries with important access to commercial markets may not be interested in preferential loans – especially as I4H loans would remain tied to conditions, potentially restricting countries' freedom to use the money as they wish. On the opposite end of the spectrum, countries with important external debt might not be eligible for additional loans, which may threaten their financial stability. Therefore, customized incentive packages may include preferential access to intellectual property rights for drugs and medical devices, technological transfers, work visas, scholarships and educational opportunities, debt relief, and in‐kind exchanges of natural resources.
- Incentive packages are developed on a case‐by‐case basis, depending on each country's situation and needs. To ensure equity, the guiding principles mentioned above remain. The financial value of each package will also be calculated; for example, if scholarships and student visas are considered as part of an incentive package, their current and future financial value (in terms of added human capital) are computed, and compared to the value of a preferential loan. In this scenario, the I4H Alliance is responsible for brokering negotiations between incentive‐providing countries or agencies and recipients. Although we acknowledge the complexity of such a design, these packages can appeal to countries that may not be attracted to loans.

We envision the I4H Alliance as an independent body, with its own governance principles and structure. Strong principles of country ownership, South‐South collaboration, transparency, and accountability should underpin its governance modality, which includes: 1) a multistakeholder, representative board; 2) an executive office; 3) one strategy committee on health target planning and evaluation, and one strategy committee on incentives; 4) a series of country coordination teams that report to the executive office and that liaise between the strategy committees and the participating countries; and 5) an auditing office that coordinate process auditing for the countries' health plans and annual progress reports. Besides, we also foresee the I4H Alliance may serve as a platform for coalition building between countries with similar experiences and complementary resources.

## 5 Incentives for Health (I4H) in Practice: A Hypothetical Case Study of Vietnam

We return to the example of Vietnam for a case study on how I4H may promote health financing in a participating country. Vietnam's GNIpc was $1 980 USD in 2015.^23^ The country currently has access to IDA concessional loans, but is set to lose access by the end of 2017. Under the leadership of its Ministry of Finance and Ministry of Health, Vietnam may decide to enter the I4H Alliance with a health plan focused on reducing road injury, currently the second cause of premature deaths in the country (GBD 2010).^30^ This five‐year health target will seek to reduce its road traffic death rate from 24.5 per 100 000 in 2013, to 15 per 100 000 by 2022, an objective in line with SDG target 3.6 (to “halve global deaths from road traffic accidents by 2030”).

In order to achieve this, Vietnam will develop a detailed implementation plan, which includes the launch of road safety campaigns and a change in road regulations. Putatively, Vietnam estimates these activities will cost $50 million USD, which will be financed through a new traffic infraction fine. This plan will be officially agreed upon by the I4H Alliance Board, which, in collaboration with an established development financial institution (cf. below), will reward Vietnam with an incentive of $80 million USD, in a preferential loan to be used towards infrastructure projects for Vietnam's sustainable development (with no requirement that the loan be spent on health). In our more advanced model, this preferential loan could be replaced in part by scholarships financed by China and South Korea to support overseas study for Vietnam's growing student population.

Between 2017 and 2022, Vietnam will implement the plan it has set out for itself, and report its efforts through the M&E framework agreed upon at the project's start. In 2022, once Vietnam successfully achieves its health target, it will receive the preferential loan, set for a three‐year roll‐out starting in 2023. In our more advanced model, Vietnam gains access to overseas scholarships, scheduled between 2022 and 2032.

## 6 Benefits of the I4H Alliance

Improving health outcomes in MICs is a highly complex issue, and no single intervention will resolve it entirely. As such, this proposal is designed as a complement to ongoing initiatives, in order to address some of the key shortcomings of the current DAH system design.

First, the I4H model addresses the currently unfulfilled needs and capacities of MICs by involving them both as recipients and donors. As recipients of I4H incentives, MICs retain control and ownership over their health investments, while achieving outcomes that they and the global health community have determined are important. As donors, the opportunity to be a contributor of incentives can be a platform for inclined UMICs to launch their aid aspirations. The success of I4H is therefore integrally tied to MIC participation, and our explicit coalition‐building strategies will further promote collaboration among MICs.As well, the I4H Alliance addresses some of the challenges related to the interdependence that exists between health and other sectors, such as trade or agriculture, by linking health with other areas of sustainable development. Since the deployment of I4H incentives are not restricted to further investments in health, it provides Ministries of Health with leverage to push for more integrated health interventions that will more easily garner support from Ministries of Finance and Development.

Next, as I4H's investment priorities are explicitly linked to multi‐dimensional criteria, the Alliance addresses the disconnection between prevailing metrics and needs. By explicitly tying incentives to health needs, we ensure DAH allocation moves away from the typical GNIpc‐based approach. Furthermore, as I4H's target setting mechanism includes equity considerations, it can help address some of the most currently pressing and underinvested health needs in MICs.

Regarding the tensions and questions around accountability for DAH and health in MICs, the above‐mentioned ownership and central role of MICs should offer some hope for progress. Careful attention has also been paid to the I4H Alliance's proposed governance structure, to ensure clear accountability as well as fair representation of MICs and their peoples as chief stewards of the enterprise.

## 7 Towards an Integrated Approach to Global Challenges

Since 2012, much has been discussed about the “end of the golden age” for global health financing.^31^ As DAH worldwide continues to stagnate in recent year, and debates turn from expanding total global health funding towards increasing efficiency and value for money, is now really the right time to be advocating for the creation of a new global health mechanism?

We believe that the answer is yes. As international funding for health is becoming limited, a mechanism such as the I4H Alliance can help countries transition towards more sustainable sources of domestic funding, while leveraging the total (non‐health specific) capacities of new donors. This combination is likely to appeal to current bilateral and multilateral donors as a viable solution to concerns around the sustainability of traditional DAH. As well, recipient countries may welcome I4H as a way to both move away from dependency on HIC‐mediated DAH, and gain access to preferential financing to sustain their economic growth.

Specifically, we see the recent creation of the BRICS‐led New Development Bank (NDB) as a window of opportunity to launch the I4H Alliance. While we believe that the I4H Alliance has potential as a stand‐alone institution or as part of a more long‐established financial organization (such as the World Bank or a regional development bank), housing it at the NDB could incentivize the BRICS countries to play an increased role among MICs in global health. As well, while the health and policy impacts of I4H can be enormous, its implementing costs should remain relatively low. Our initial calculations project an annual operating budget between $13 million and $160 million USD/year at scale, for loan incentives valued at $1.5 billion USD per year. This level of funding should be attainable, especially considering the long‐term benefits associated with increased domestic sustainability triggered by the mechanism.

Our proposal is a radical departure from the status quo. Beyond global health, our hope is to break traditional silos too often perceived as immutable: between Ministries of Health and other Ministries, between low‐income, middle‐income, and high‐income countries, or between health and other public goals. Indeed, while the I4H Alliance was developed with global health in mind, we believe this approach could ultimately be used to address other challenges such as climate change or water shortage. Overall, the I4H Alliance can be a powerful mechanism to increase spending and programming on important social issues, encourage South‐South collaboration, and ultimately bring about global progress towards the SDGs. Now more than ever, new and innovative approaches are needed to ensure that truly global challenges are addressed in effective, interdependent ways.

## Conflict of Interest

The authors declare no conflict of interest.
